# Supplementary material for: Quantifying HiPSC-CM structural organization at scale with deep learning-enhanced SarcGraph
Source: PLoS Comput Biol. 2025 Oct 3;21(10):e1013436. doi: 10.1371/journal.pcbi.1013436 (PMC12520406; doi:10.1371/journal.pcbi.1013436)
Supplement: S1 Appendix — Contains Appendices A–G, Figs A1–A12, and Tables A1–A2. (PDF) [file pcbi.1013436.s001.pdf]

Quantifying HiPSC-CM Structural Organization at Scale with Deep Learning-Enhanced SarcGraph

S1 Appendix · Extended methods, results, and comparisons

A Model Training Details

A.1 Representation Learning with SimCLR

We trained a self-supervised representation learning model using the SimCLR framework with an EfficientNetV2-S backbone, where the classification head was removed to retain a 1024-dimensional feature representation. A projection head was added on top, consisting of two layers with ReLU activations and a final linear layer that projects the output to a 128-dimensional embedding vector.

The model was trained with NT-Xent loss using the LARS optimizer (learning rate = 1.0, momentum = 0.9, weight decay = 10^{-4}). Training lasted 200 epochs with gradient accumulation (step size = 4). The learning rate followed a linear warm-up for the first 10 epochs, reaching the base learning rate, and then decayed following a cosine annealing schedule. A temperature parameter of 0.1 was used in the contrastive loss function. Training was conducted using SyncBatchNorm for improved multi-GPU training, with varying GPU models in both single-GPU and distributed multi-GPU parallel training setups. Training was performed with 16-bit precision.

SimCLR relies on contrastive learning, which requires generating different augmented views of the same image. To achieve this, we randomly applied a combination of transformations, including random resized cropping, Gaussian blurring, and normalization. The training batch size was set to 512 for two-GPU setups. For single-GPU training or training with four GPUs, the batch size and gradient accumulation step size were adjusted to ensure that weight updates were always performed after processing 1024 samples through the model.

A.2 Fine-Tuning with Labeled Data

For fine-tuning, we initialized an EfficientNetV2-S model with pretrained SimCLR weights and replaced the projection head with a classification head. Instead of discarding the entire projection head, we retained its first layer, including the linear transformation and ReLU activation, resulting in a 512-dimensional input to the classification head, which mapped the features to two output classes.

The model was trained using the LARS optimizer (learning rate = 0.01) with a cosine annealing scheduler and gradient accumulation (step size = 2) over 100 epochs. The loss function used was binary cross-entropy with logits, scaled by a temperature parameter of 0.1. Training was performed on a single GPU using 16-bit precision.

Data augmentation included random resized cropping, horizontal flipping, and normalization. These augmentations were used both for regularization and to introduce transformations similar to those used in the representation learning phase. Additionally, we expanded the dataset using rotations (90°, 180°, 270°) and horizontal flips. The dataset was split into 80% training and 20% validation.

Models were saved based on the best validation loss, and training was performed 10 times with different random seeds. An ensemble of 8 trained models was used at inference time for more robust classification.

B Graph Scoring: Global Myofibril Alignment

This Section describes the procedure for graph scoring using the Global Myofibril Alignment method introduced in the Methods section. The method proceeds in four stages: score initialization, iterative partial-score updates, aggregation of partial scores, and score normalization.

B.1 Initialization

Each edge $e = (u, v)$ maintains two partial scores, one from each endpoint's perspective, denoted by $s_u(e)$ and $s_v(e)$. Both partial scores are initialized to 0. Altogether, each edge e has three main quantities:

- score(e): the primary alignment score to be computed.
- $s_u(e) = 0$: partial score from node u's perspective.
- $s_v(e) = 0$: partial score from node v's perspective.

B.2 Iterative Partial-Score Updates

We perform $T = 6$ iterations of the update process. In each iteration, for every edge $e = (u, v)$, we perform two updates: one to compute $s_v(e)$ ("from u to v"), and another to compute $s_u(e)$ ("from v to u"). Below, we describe the update for $s_v(e)$; the other update follows analogously.

Neighborhood Alignment. Let $\alpha_{(u,v),(v,w)}$ be the angle between edges (u, v) and (v, w). If $\alpha_{(u,v),(v,w)}$ is smaller than a chosen threshold (30°), we say that (v, w) is aligned with (u, v).

Partial Score Update. For each aligned neighbor (v, w), define:

$$C_{(v,w)} \;=\; \cos\bigl(\alpha_{(u,v),(v,w)}\bigr) \;\times\; \Bigl(1 \;+\; s_w(v, w)\Bigr) \tag{1}$$

We take the maximum of all such contributions and multiply it by a scaling factor $\eta = 0.8$. Hence, the updated partial score at v for edge (u, v) is

$$s_v(e) \;=\; \eta \;\times\; \max_{(v,w)} \Bigl\{ C_{(v,w)} \Bigr\} \tag{2}$$

This step is then repeated in the reverse direction to obtain $s_u(e)$ using the neighbors of u.

B.3 Aggregation of Partial Scores

After T iterations, the combined (pre-normalized) score of edge $e = (u, v)$ is computed as the average of its two partial scores:

$$\text{score}(e) \;=\; \frac{s_u(e) \;+\; s_v(e)}{2}. \tag{3}$$

Both endpoints' perspectives thus contribute to the final alignment score of the edge.

B.4 Normalization

Let

$$S_{\max} \;=\; \min\Bigl(T + 1,\; \max_{e \in E}\bigl\{\text{score}(e)\bigr\}\Bigr) \tag{4}$$

We then normalize each edge's score by $S_{\max}$ so that the final value falls between 0 and 1:

$$\text{score}(e) \;=\; \frac{\text{score}(e)}{S_{\max}} \tag{5}$$

Edges that has the potential to form longer myofibril structures retain higher normalized scores.

C Features Distribution Across Datasets

This appendix provides further visualization of feature distributions referenced in Section 4.5.1. We present three sets of histograms (Fig A1, Fig A2, and Fig A3), each containing the distribution of three selected features across all datasets (Train, Test FISH, and Test Live). The cell organization categories (low, medium, and high) are defined by average expert scores (≤ 2 for low, ≥ 4 for high, and the remainder as medium).

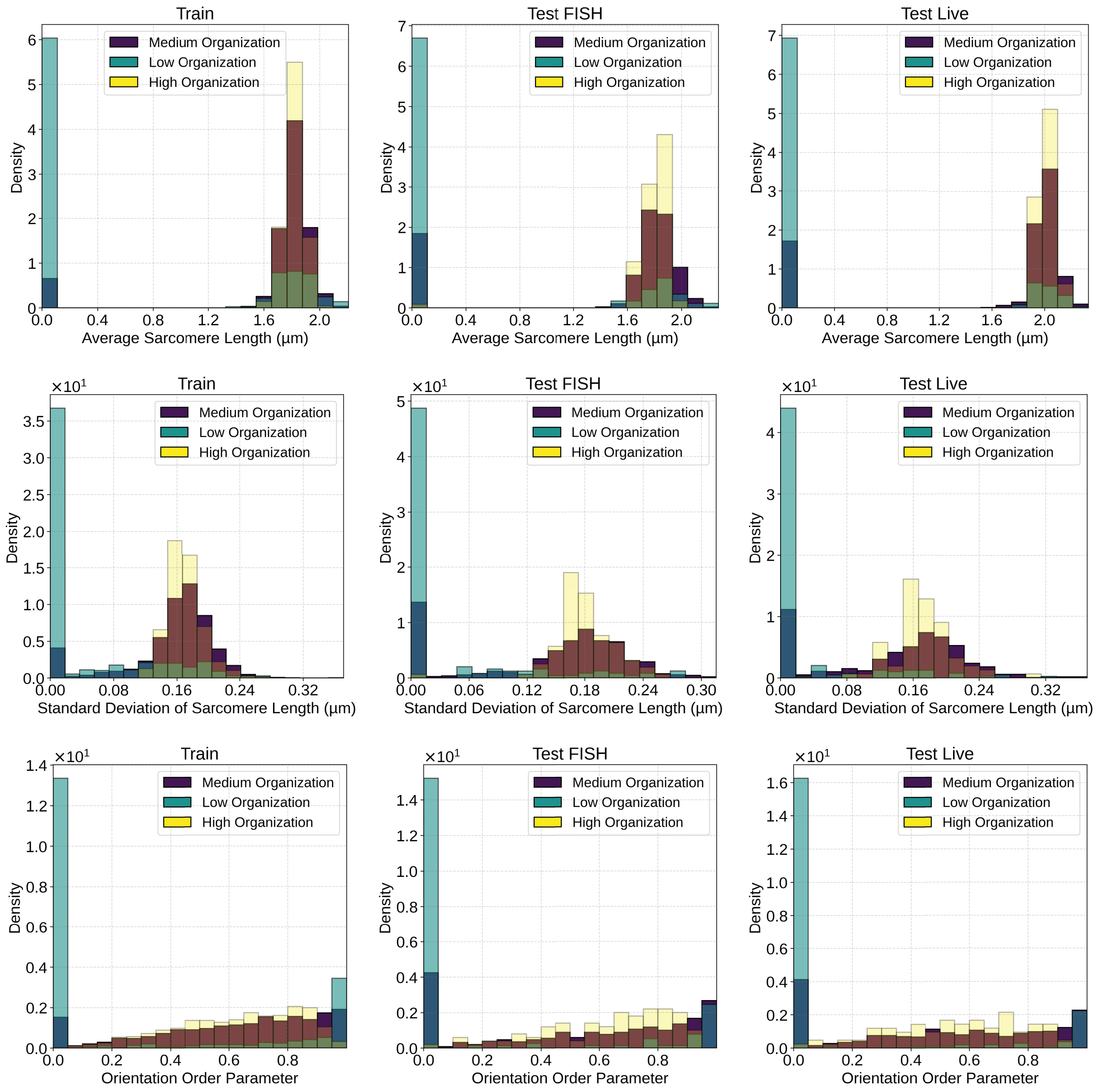

Fig A1: Distribution of average sarcomere length, standard deviation of sarcomere length, and orientation order parameter across Train, Test FISH, and Test Live datasets.

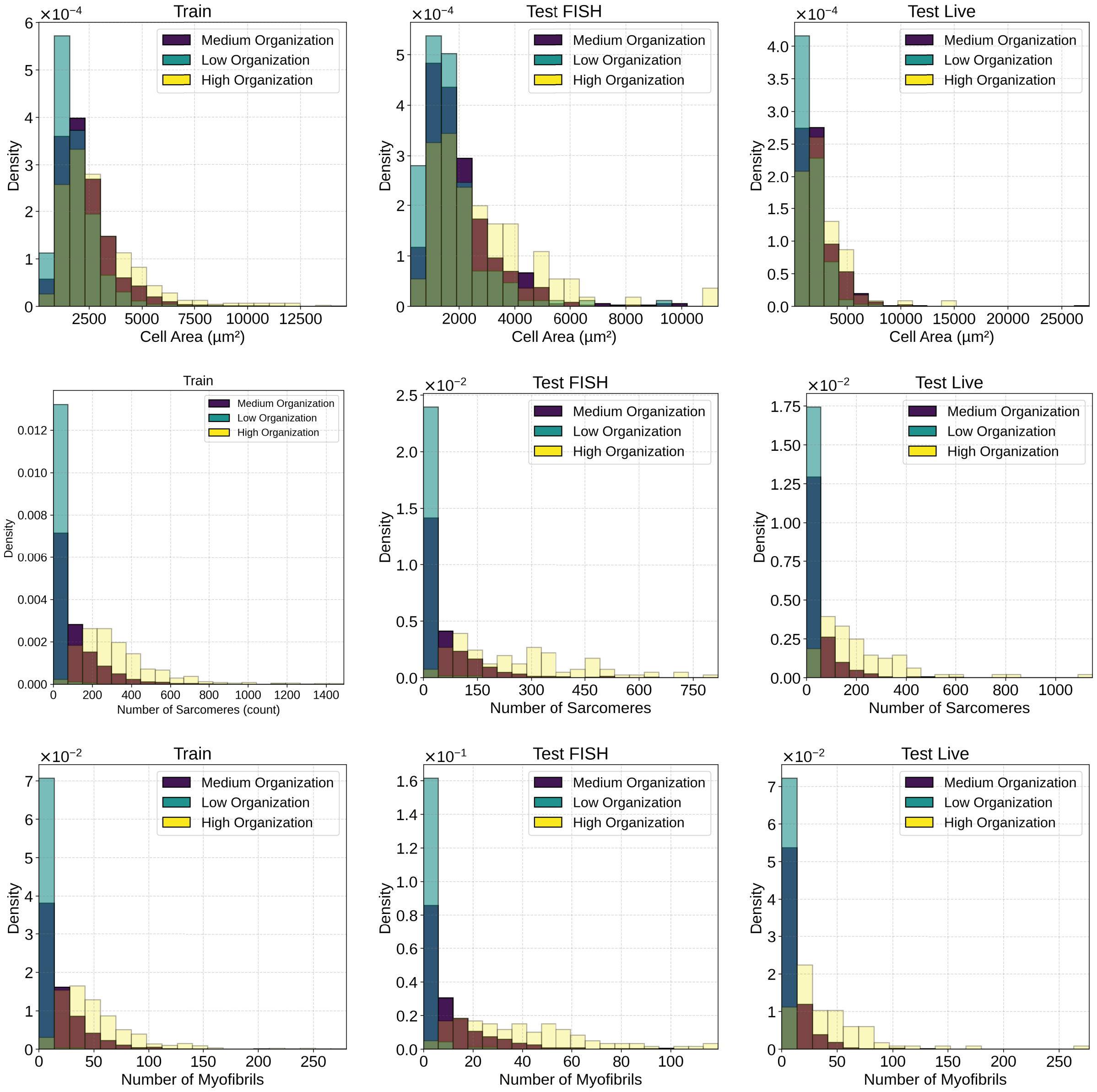

Fig A2: Distribution of cell area, number of sarcomeres, and number of myofibrils across Train, Test FISH, and Test Live datasets.

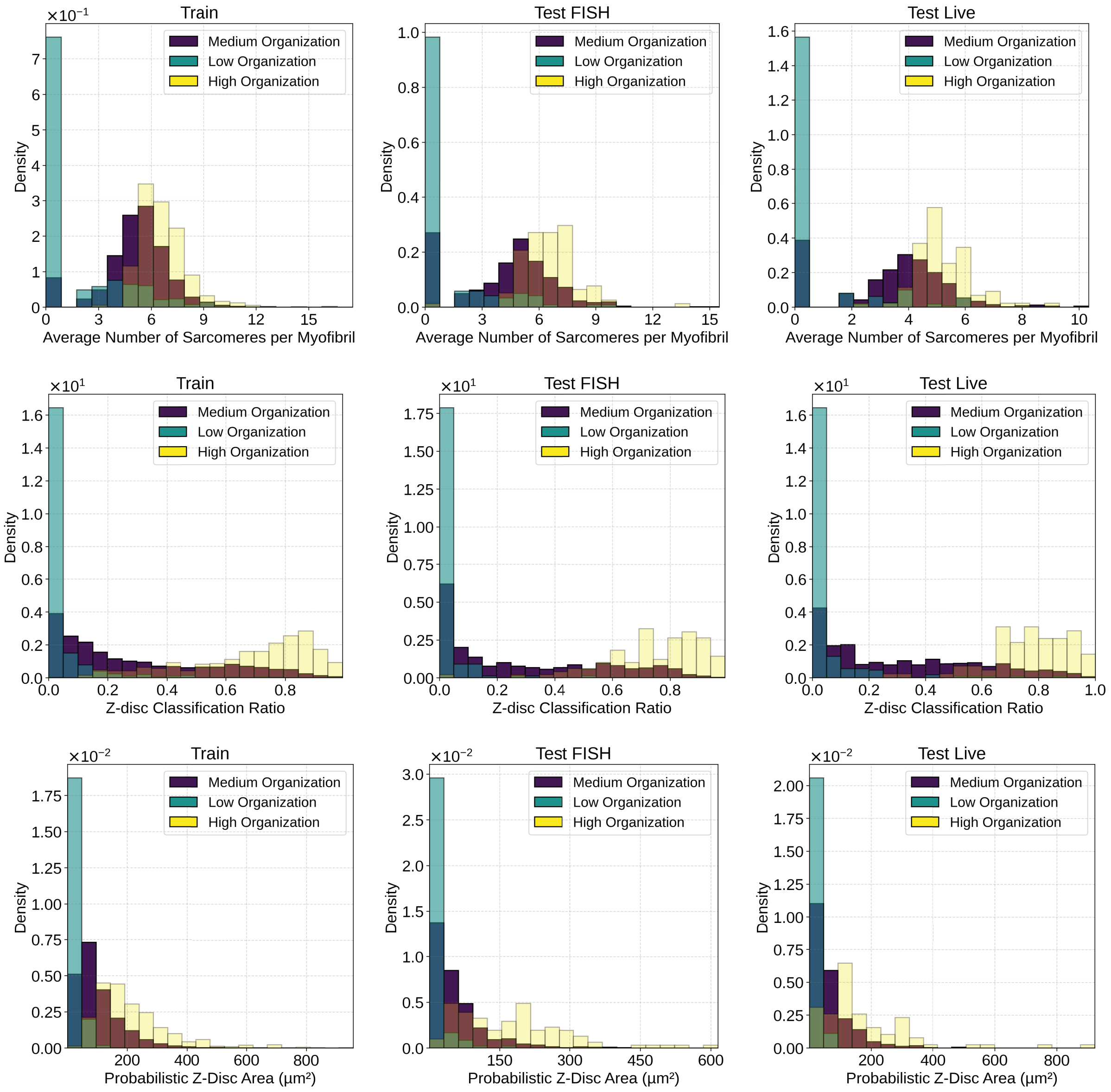

Fig A3: Distribution of average number of sarcomeres per myofibril, z-disc classification ratio, and probabilistic z-disc area across Train, Test FISH, and Test Live datasets.

D Additional Cell Visualizations for Analysis of Model Performance

This appendix provides additional visualizations to support the results from Sections 5.3 and 5.4. Fig A5, Fig A6, and Fig A7 include sample images from the Train, Test FISH, and Test Live datasets, respectively, along with their expert-assigned organization scores, SVR-predicted scores, and decision tree clustering labels (low, medium, or high organization). These examples illustrate both accurate and inaccurate model predictions. Additionally, Fig A4 shows histograms of SVR-predicted score distributions for cell organization categories determined by average expert scores (≤ 2 for low, ≥ 4 for high, and the remainder as medium).

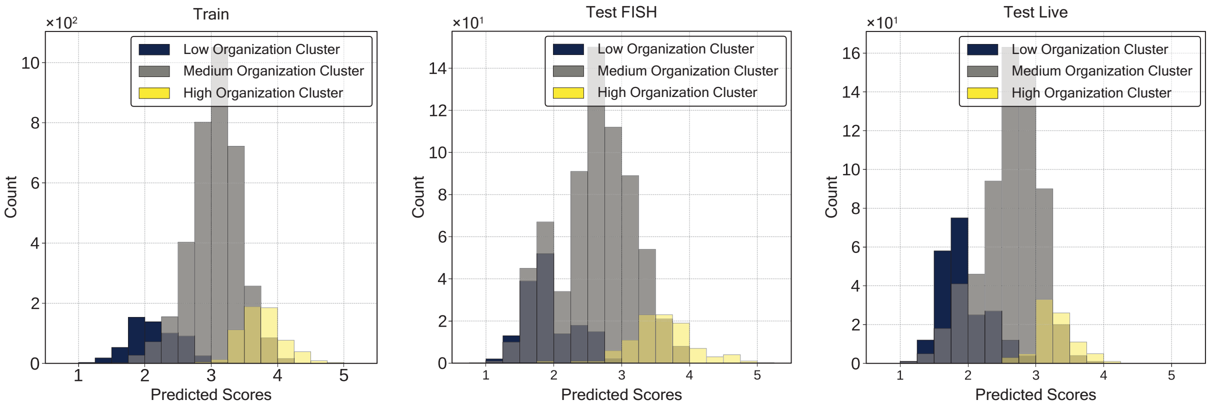

Fig A4: SVR predicted score distributions for low, medium, and high organization categories in Train, Test FISH, and Test Live datasets.

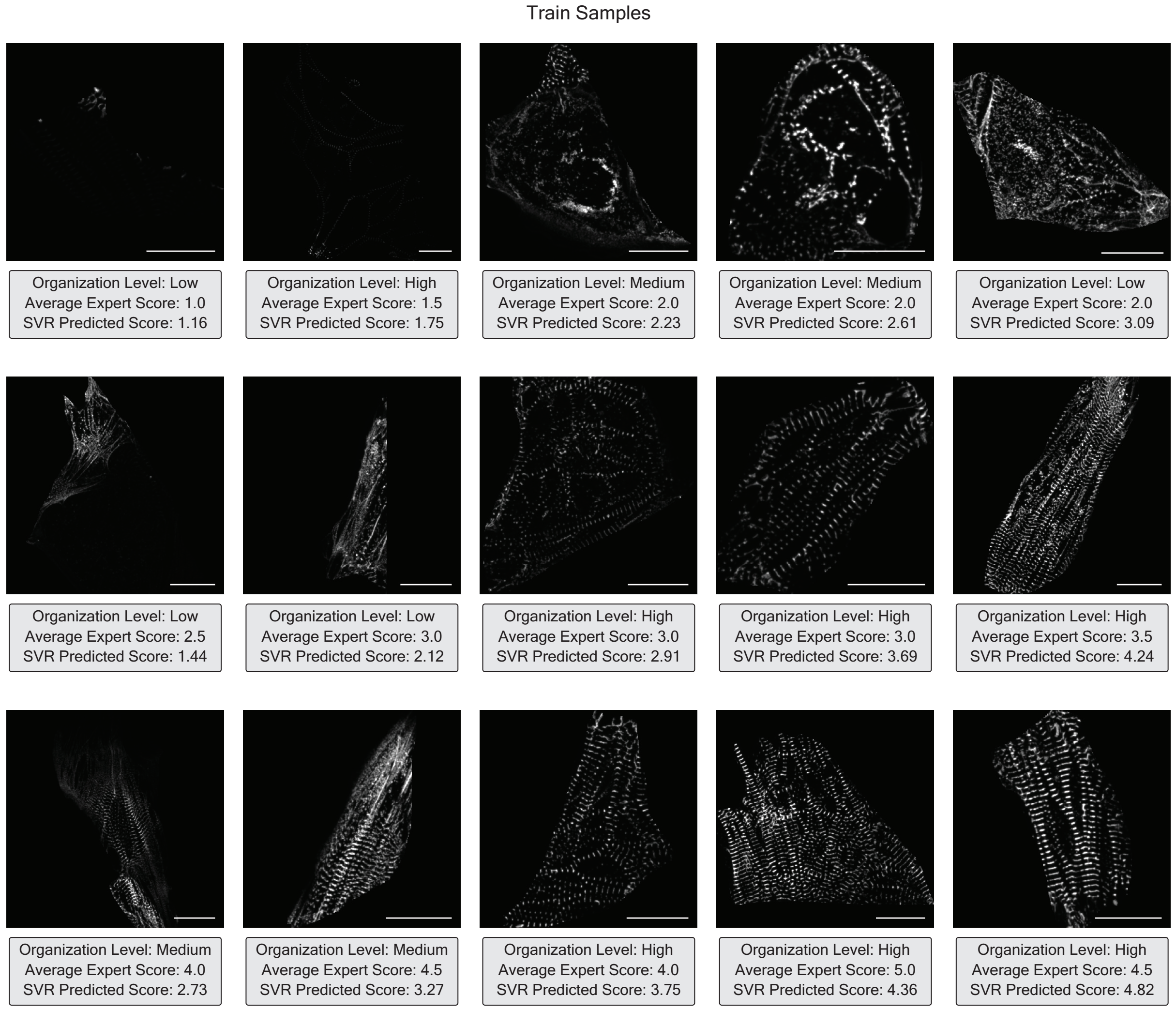

Fig A5: Sample cell images from the Train dataset visualized with expert scores, SVR predicted scores, and decision tree assigned categories.

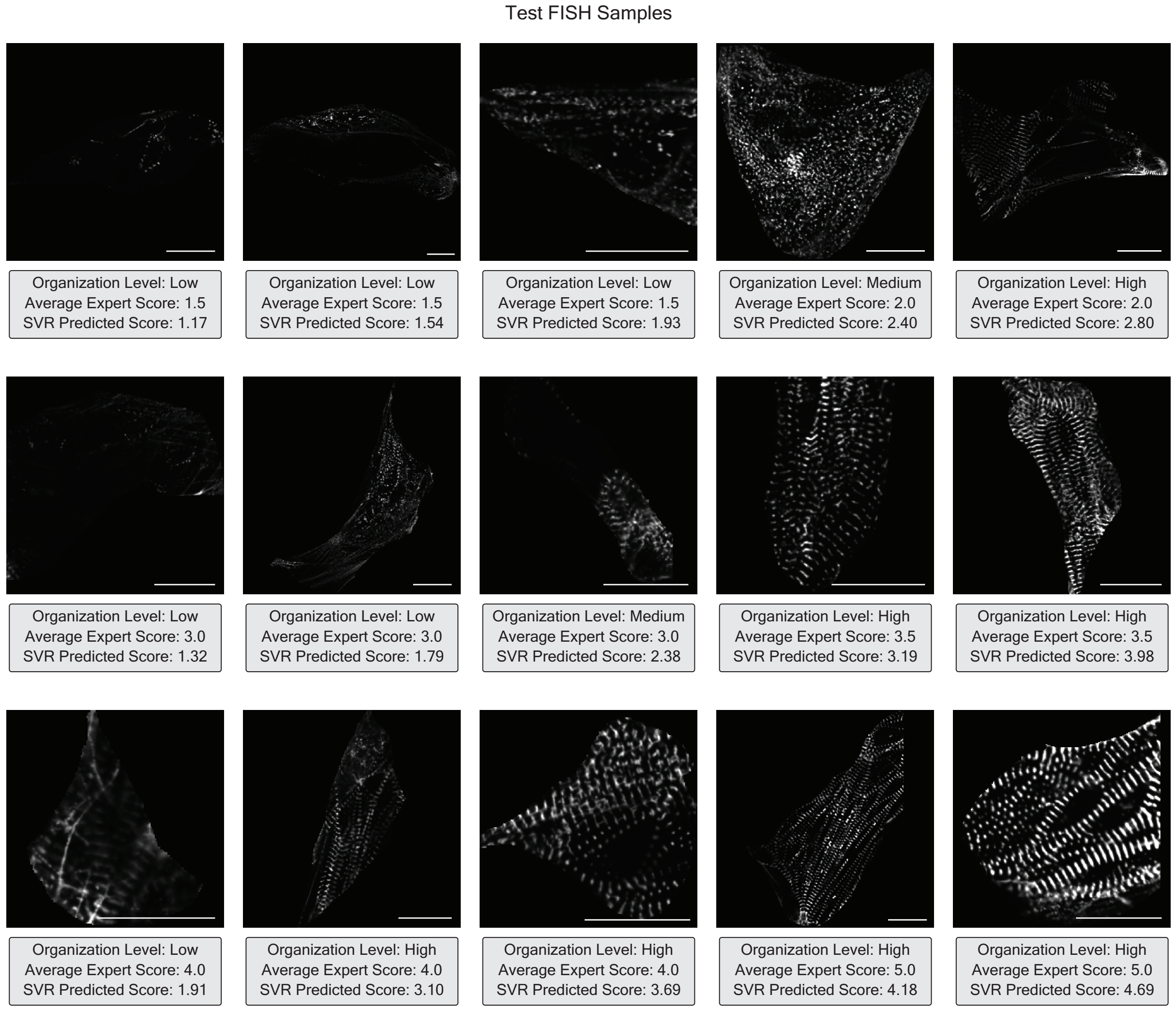

Fig A6: Sample cell images from the Test FISH dataset visualized with expert scores, SVR predicted scores, and decision tree assigned categories.

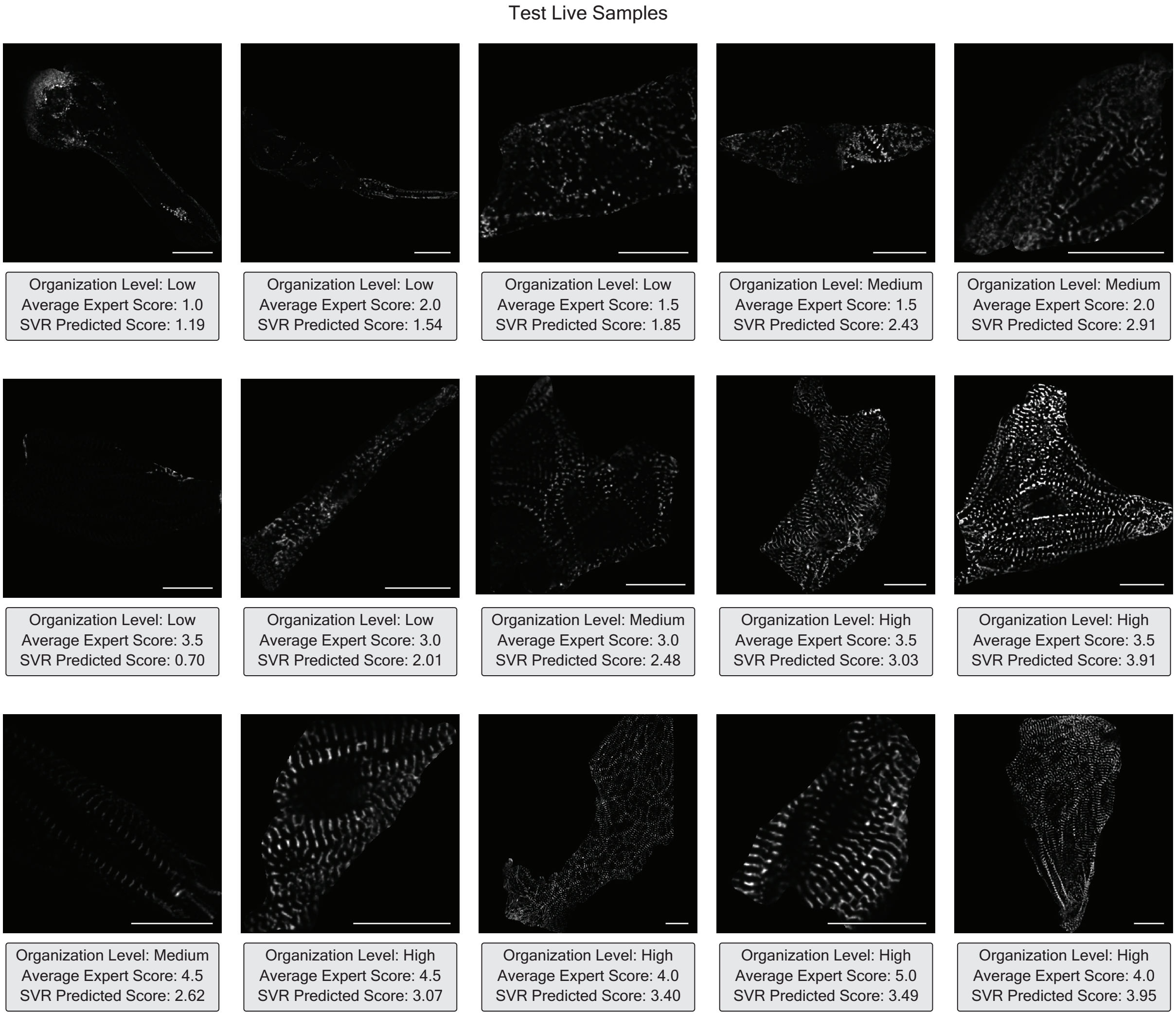

Fig A7: Sample cell images from the Test Live dataset visualized with expert scores, SVR predicted scores, and decision tree assigned categories.

E Z-disc Location Correction Pipeline

In Section 4.3.2, we highlighted limitations in the original SarcGraph algorithm for locating z-disc centers from segmented contours and proposed a new intensity-based method to address these issues. Specifically, we showed that relying on geometric centroids often misplaces z-disc locations, especially when contours merge multiple z-discs. This was illustrated in Fig 2 panel D. Here, we provide a visual overview of the proposed correction pipeline in Fig A8.

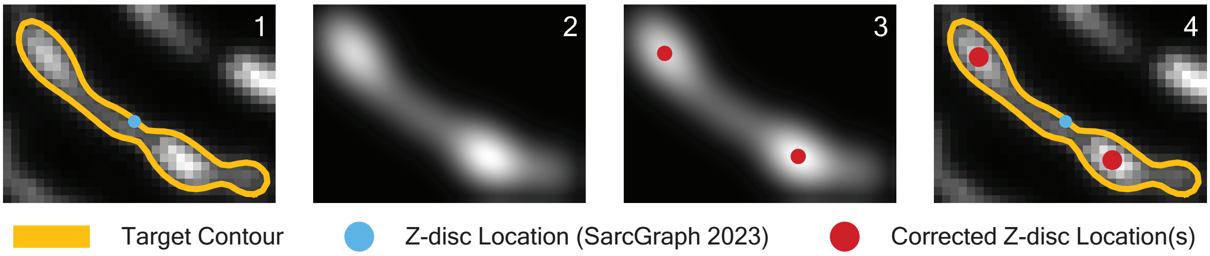

Fig A8: Z-disc center correction pipeline. (1) Target contour from segmentation output, with original SarcGraph z-disc center shown as a blue dot (geometric centroid). (2) Interpolated and smoothed pixel intensity within the target contour. (3) Local maxima identified via gradient-based optimization with multiple random starts with red dots. (4) Comparison of original and corrected z-disc centers.

F Extended Visualization of Scoring Mechanisms

In Section 4.4.1 and Fig 3 panel C, we introduced four distinct graph-scoring mechanisms. Here, we provide a single, enlarged, side-by-side comparison of the same graph scored under each method to clarify which structural features each algorithm emphasizes (Fig A9).

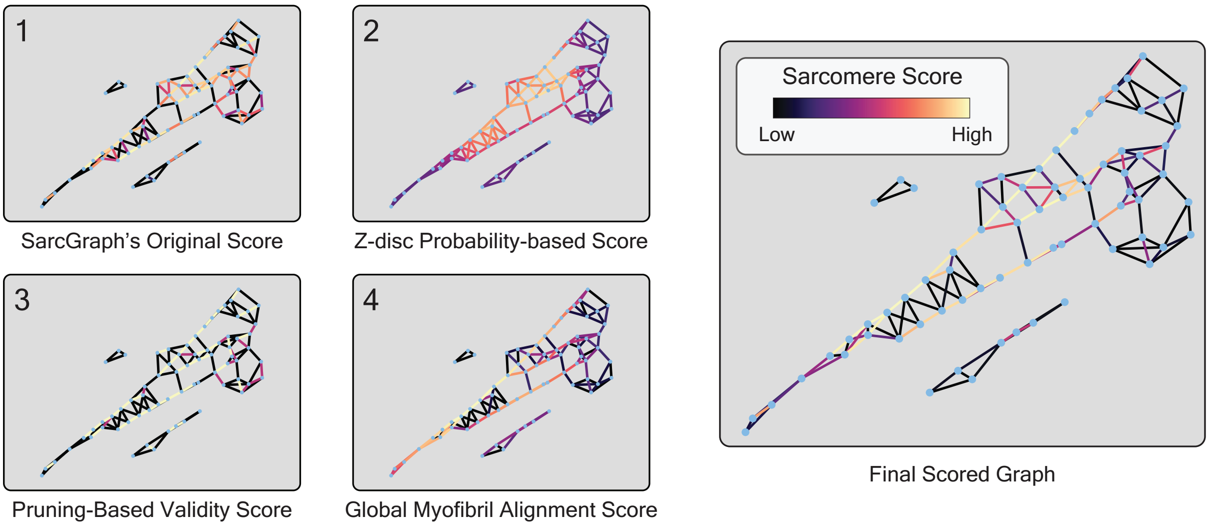

Fig A9: Extended comparison of SarcGraph graph-scoring mechanisms: (1) original SarcGraph scoring; (2) z-disc probability-based scoring; (3) pruning-based validity scoring; (4) global myofibril alignment scoring.

G Comparing SarcGraph Against Existing Methods

This Appendix focuses on the comparative benchmarking of our modified SarcGraph pipeline and the rationale behind certain methodological comparisons. Our primary objective in this work is to improve SarcGraph's robustness, particularly in handling immature or structurally disordered cells, and to effectively filter out non-sarcomeric structures. We demonstrate our pipeline's generalizability and improved performance through a direct comparison with SarcOmere Texture Analysis (SOTA) [6] on an independent dataset published along with their paper. Furthermore, we clarify why direct comparisons with other established tools are not always informative, as many operate with different methodological goals (e.g., focusing on tracking over time or global textural features rather than detailed individual sarcomere detection).

Benchmarking SarcGraph Against SOTA

Evaluating SarcGraph's performance against leading tools on external data is crucial. This comparison serves two key purposes. First, to showcase the significant improvements of the new SarcGraph version over the version published in 2021 [8] (SarcGraph 2021) and the version published in 2023 [2] (SarcGraph JOSS), particularly in accurately computing average sarcomere length through enhanced sarcomere detection. Second, to demonstrate the generalizability of our deep learning-based Z-disk classifier on a previously unseen dataset, implemented without any fine-tuning. To this end, we performed a direct comparison with SOTA, utilizing a publicly available dataset that was originally published alongside the SOTA paper [6].

A key challenge in this cross-dataset application involved differences in image characteristics, such as brightness, to which some aspects of our pipeline can be sensitive especially since the SOTA dataset images are post-processed. Despite these varying experimental conditions and post-processing, SarcGraph performed remarkably well, demonstrating promising generalizability.

Fig A10 presents visualizations of sarcomere detection by SarcGraph on representative SOTA samples, showcasing its ability to effectively distinguish Z-discs from other structures in a completely new context. Though there were some false positives and negatives in the final sarcomere detection results, we believe that even without additional fine-tuning the results were quite good. Quantitatively, Table A1 compares the average sarcomere lengths computed using the modified SarcGraph (this work), SarcGraph 2021, SarcGraph JOSS, and SOTA. Notably, the modified SarcGraph's sarcomere length measurements are more comparable to SOTA's results than those from the other versions of SarcGraph. This improvement is primarily due to our modified pipeline's ability to largely suppress spurious detections that occurred in the other versions, along with its overall enhanced sarcomere detection capabilities, leading to more accurate and reliable sarcomere length estimations.

Table A1: Comparison of average sarcomere length (μm) computed with SOTA, SarcGraph 2021, SarcGraph JOSS, and the modified SarcGraph (this work) for selected samples from the SOTA dataset.

		**SarcGraph 2021**		**SarcGraph (JOSS)**		**Modified SarcGraph**	
**Image #**	**SOTA**	**mean**	**median**	**mean**	**median**	**mean**	**median**
60	2.09	1.60	1.76	2.23	2.20	2.14	2.10
08	2.17	1.49	1.46	2.25	2.25	2.18	2.16
31	2.08	1.44	1.37	2.23	2.19	2.12	2.11
11	1.83	1.36	1.38	2.17	2.10	1.95	1.87

Benchmarking SarcGraph Against SarcAsM

SarcAsM (Sarcomere Analysis Multitool) is the method that shares the highest conceptual resemblance to our SarcGraph approach [1]. As a brief note, the SarcAsM pre-print was posted

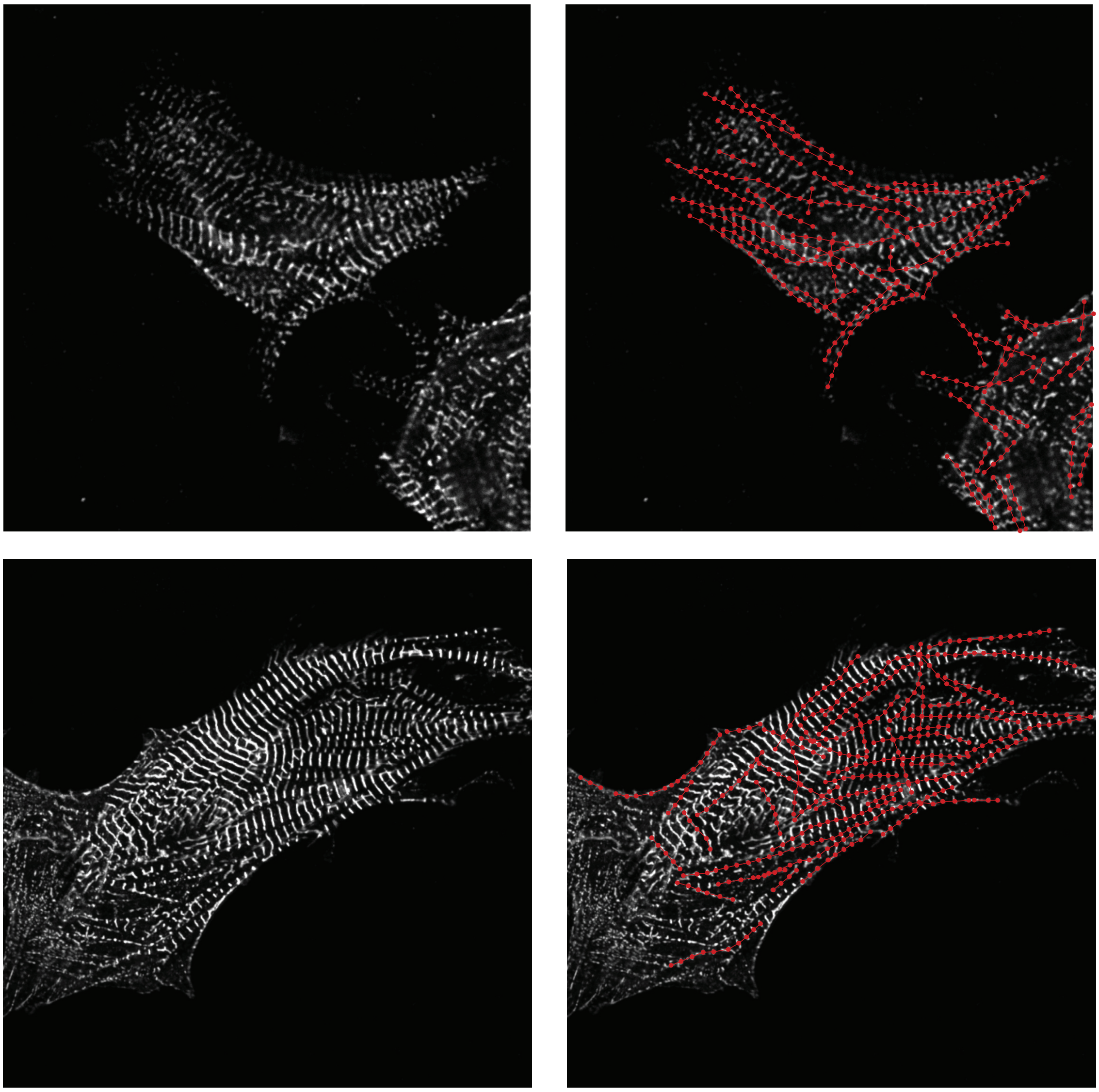

Fig A10: Visual comparison of SarcGraph sarcomere detection on representative samples from the SOTA dataset (top: sample 08, bottom: sample 11).

on May 4th 2025, approximately three months after we submitted this work. SarcAsM employs deep learning-based segmentation to identify Z-discs, which are then used by graph-based methods to identify potential myofibrils. Once z-discs are segmented and sarcomere and myofibril analysis is completed SarcAsM outputs a comprehensive list of structural features. The authors provide SarcAsM as an open-source Python package and a stand-alone application, facilitating community access and use.

In Fig A11 and Fig A12, we visually compare the sarcomere detection results for five representative samples from the Allen Institute dataset used in this work, with each sample representing a score group from 1 to 5. For each score group, the image on the left shows the analysis results using SarcAsM (where cyan color showcases segmented Z-discs and shades of red indicate potential myofibrils generated through their graph-based method). The visualizations reveal a very good agreement between sarcomeres detected with SarcGraph and SarcAsM, with this agreement being particularly noticeable in samples with a large number of sarcomeres. For samples with very few sarcomeres, while SarcAsM's myofibril detection generally matches ours visually, its output values for average sarcomere length and number of M-bands (used as a proxy for sarcomeres) are believed to be less accurate compared to SarcGraph's results. To quantitatively compare the two methods, we present the average sarcomere length, number of detected sarcomeres (using SarcAsM's number of M-bands as a proxy for its count), and sarcomere standard deviation between SarcGraph and SarcAsM in Table A2.

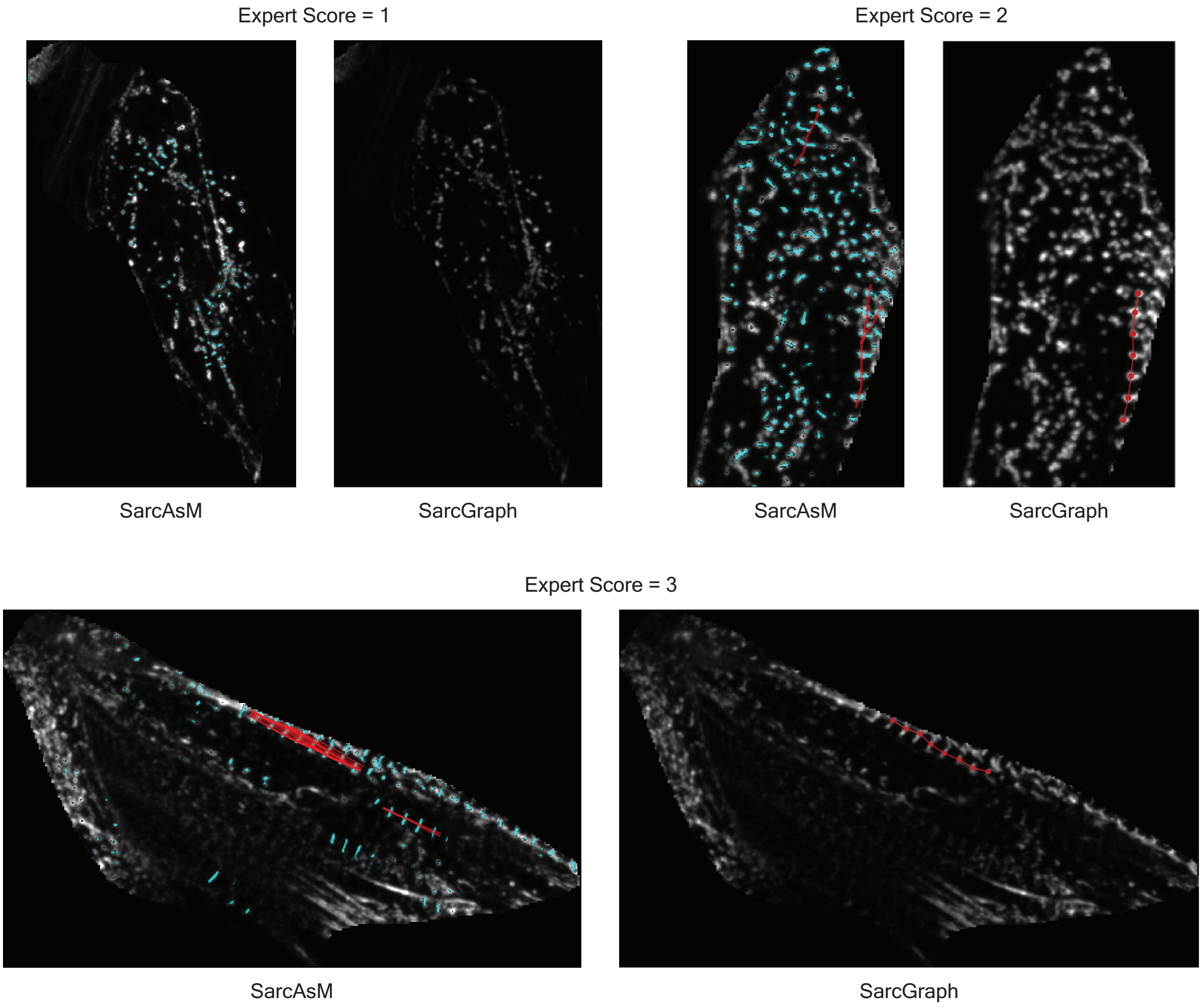

Fig A11: Visual comparison of SarcGraph vs. SarcAsM sarcomere detection in representative cells with expert scores 1-3. SarcAsM shows segmented Z-discs (cyan) and myofibrils (red).

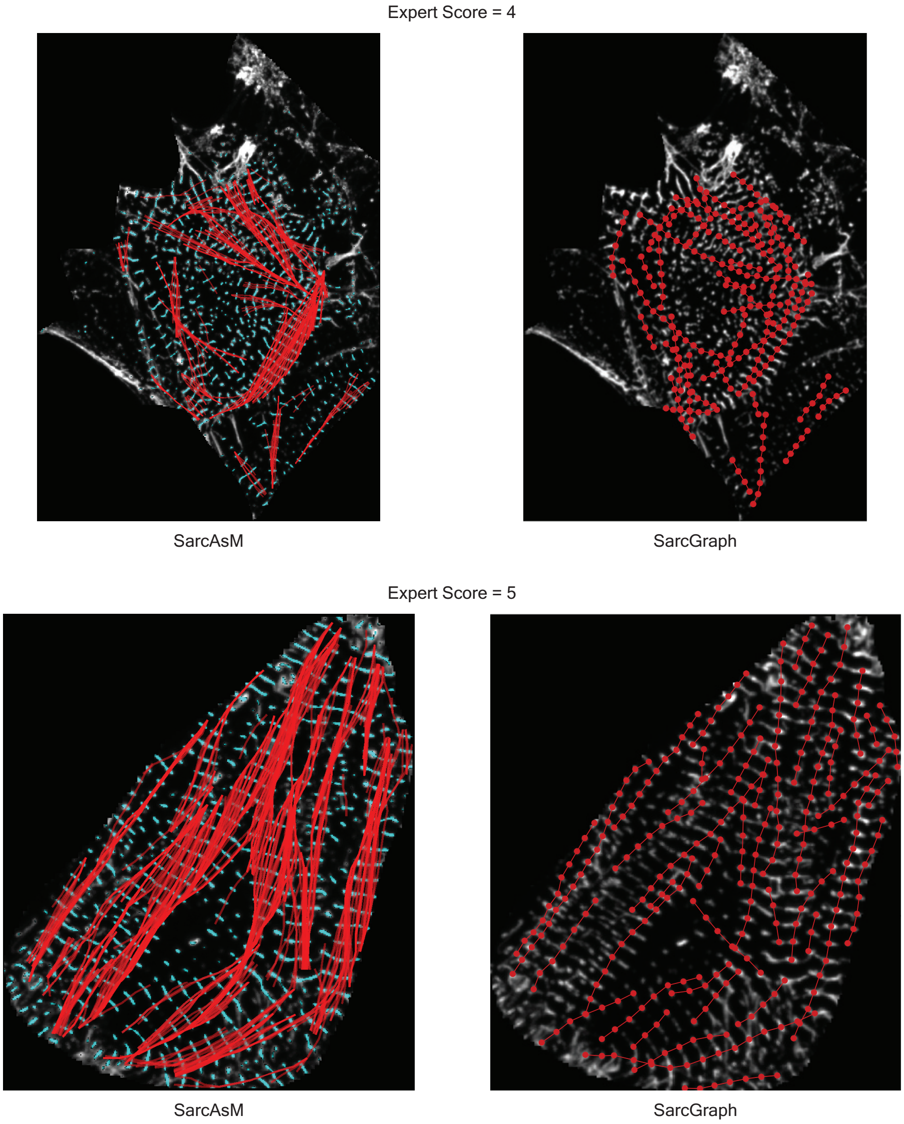

Fig A12: Visual comparison of SarcGraph vs. SarcAsM sarcomere detection in representative cells with expert scores 4-5. SarcAsM shows segmented Z-discs (cyan) and myofibrils (red).

Table A2: Comparison of sarcomere features computed by SarcGraph and SarcAsM for 5 samples across different expert score groups. *Number of sarcomeres for SarcAsM refers to the number of m-bands detected by the tool.

	Average Sarcomere Length (μm)		STD Sarcomere Length (μm)		Number of Sarcomeres*	
	**SarcAsM**	**SarcGraph**	**SarcAsM**	**SarcGraph**	**SarcAsM**	**SarcGraph**
**Expert Score 1**	1.64	-	0.42	-	18	0
**Expert Score 2**	1.68	1.9	0.34	0.09	60	6
**Expert Score 3**	1.77	1.78	0.23	0.04	43	7
**Expert Score 4**	1.83	1.86	0.26	0.21	278	252
**Expert Score 5**	1.81	1.83	0.17	0.16	250	282

Considerations for Other Benchmarking Tools

While rigorous, tool-to-tool comparisons are valuable, we have focused our benchmarking efforts on areas most relevant to the novel contributions of this manuscript. Tools such as Contrax [4], SarcOptim [5], and zlineDetection [3] generally operate under different methodological goals or are optimized for specific types of data that do not align with the unique challenges addressed by our modified SarcGraph pipeline. For instance, Contrax is designed to measure traction force, which is unavailable in our dataset, while SarcOptim and ZlineDetection are predominantly designed for mature cells with well-defined and often contracting sarcomeres.

Crucially, tools like SarcTrack [7] and earlier iterations of SarcGraph (e.g., [8], [2]) focus primarily on tracking sarcomeres over time in video sequences of beating cells. In contrast, our key focus in this work is on single, static images, and the modified SarcGraph pipeline is designed to accurately distinguish Z-discs from other structures and to robustly process images of diverse cell maturity, especially immature cells where true sarcomeric structures are sparse or ambiguous. Therefore, direct comparisons with these tools would not effectively showcase our method's advancements in these challenging contexts.

References

[1] Daniel Härtter, Lara Hauke, Til Driehorst, Yuxi Long, Guobin Bao, Andreas Primeßnig, Branimir Berečić, Lukas Cyganek, Malte Tiburcy, Christoph F Schmidt, et al. Sarcasm: Ai-based multiscale analysis of sarcomere organization and contractility in cardiomyocytes. *bioRxiv*, pages 2025–04, 2025.

[2] Saeed Mohammadzadeh and Emma Lejeune. Sarcgraph: A python package for analyzing the contractile behavior of pluripotent stem cell-derived cardiomyocytes. *Journal of Open Source Software*, 8(85):5322, 2023.

[3] Tessa Altair Morris, Jasmine Naik, Kirby Sinclair Fibben, Xiangduo Kong, Tohru Kiyono, Kyoko Yokomori, and Anna Grosberg. Striated myocyte structural integrity: Automated analysis of sarcomeric z-discs. *PLoS computational biology*, 16(3):e1007676, 2020.

[4] Gaspard Pardon, Alison S Vander Roest, Orlando Chirikian, Foster Birnbaum, Henry Lewis, Erica A Castillo, Robin Wilson, Aleksandra K Denisin, Cheavar A Blair, Colin Holbrook, et al. Tracking single hipsc-derived cardiomyocyte contractile function using contrax an efficient pipeline for traction force measurement. *Nature communications*, 15(1):5427, 2024.

[5] Côme Pasqualin, François Gannier, Angèle Yu, Claire O Malécot, Pierre Bredeloux, and Véronique Maupoil. Sarcoptim for imagej: high-frequency online sarcomere length computing on stimulated cardiomyocytes. *American Journal of Physiology-Cell Physiology*, 311(2): C277–C283, 2016.

[6] Matthew D Sutcliffe, Philip M Tan, Antonio Fernandez-Perez, Young-Jae Nam, Nikhil V Munshi, and Jeffrey J Saucerman. High content analysis identifies unique morphological features of reprogrammed cardiomyocytes. *Scientific reports*, 8(1):1258, 2018.

[7] Christopher N. Toepfer, Arun Sharma, Marcelo Cicconet, Amanda C. Garfinkel, Michael Mücke, Meraj Neyazi, Jon A.L. Willcox, Radhika Agarwal, Manuel Schmid, Jyoti Rao, Jourdan Ewoldt, Olivier Pourquié, Anant Chopra, Christopher S. Chen, Jonathan G. Seidman, and Christine E. Seidman. Sarctrack. *Circulation Research*, 124(8):1172–1183, 2019. doi: 10.1161/CIRCRESAHA.118.314505. URL `https://www.ahajournals.org/doi/abs/10.1161/CIRCRESAHA.118.314505`.

[8] Bill Zhao, Kehan Zhang, Christopher S Chen, and Emma Lejeune. Sarc-graph: Automated segmentation, tracking, and analysis of sarcomeres in hipsc-derived cardiomyocytes. *PLoS computational biology*, 17(10):e1009443, 2021.
