# Supplementary material for: Quantifying HiPSC-CM structural organization at scale with deep learning-enhanced SarcGraph
Source: PLoS Comput Biol. 2025 Oct 3;21(10):e1013436. doi: 10.1371/journal.pcbi.1013436 (PMC12520406; doi:10.1371/journal.pcbi.1013436)
Supplement: Attachment — Submitted filename: rebuttal.pdf [file pcbi.1013436.s003.pdf]

Deep Learning-Enhanced SarcGraph for Quantifying Cardiomyocyte Organization
Response to Reviewers

We would like to thank the Editor and Reviewers for their careful and constructive evaluation of our manuscript. We greatly appreciate their feedback, which has helped us improve the clarity, completeness, and impact of our work. Below, we provide a point-by-point response to each comment. Reviewer comments are shown in full, with our responses following each item. All changes made to the manuscript are highlighted in red in the revised version.

Reviewer 1 Comments and Response

This manuscript significantly advances our ability to quantify cardiomyocyte structural organization at scale by enhancing the previously published SarcGraph pipeline. Introducing a deep-learning-based z-disc classification coupled with the ensemble graph-scoring method represents an essential methodological leap forward, particularly for challenging datasets involving immature hiPSC-CMs. Its use of graph theory and deep learning methods clearly provides strong potential for generalizability beyond classical Z-disk markers. It is highly exciting and warrants publication in PLOS Computational Biology.

The primary issue with the manuscript in its current form is the accessibility and completeness of the provided GitHub repository. The repository currently lacks crucial resources, such as the pre-trained neural network models, the labeled dataset used for training, and the clearly delineated training/testing splits. While this omission may be inadvertent or intended to avoid duplication, providing direct access to all relevant datasets and models within a single, comprehensive repository would substantially improve reproducibility and uptake by the community. Additionally, enhancing the documentation to guide new users step-by-step through replicating training and prediction would make this valuable tool even more impactful.

- Thank you for your encouraging and thoughtful feedback regarding the accessibility and completeness of our GitHub repository. We completely agree that providing comprehensive resources is crucial for reproducibility and community uptake. In conjunction with re-submitting this manuscript, we have thoroughly updated the accompanying GitHub repository following this suggestion (GitHub repository). Specifically, we have added Jupyter notebook tutorials to guide users step-by-step through replicating training and prediction. And, we have created a dedicated Harvard Dataverse repository (Dataverse repository) where we have uploaded pre-trained neural network models and labeled datasets. Links to these resources are now prominently featured in the GitHub repository. We hope these additions substantially improve the accessibility, reproducibility, and overall impact of our work.

In addition, it would be great for the manuscript to add insights and discussions around the following:

1. Comparative Benchmarking: The original SarcGraph paper (Zhao et al., 2021) set a high standard by providing explicit numerical benchmarking against multiple state-of-the-art methods, synthetic datasets, and varied experimental conditions. It would be ideal for this new manuscript to perform similar explicit comparative benchmarking against leading tools such as SarcTrack, ZlineDetection, SOTA, SarcOptiM, and CONTRAX on identical datasets. Alternatively, the authors should expand the discussion to clarify why specific direct comparisons might not be informative or necessary, considering the different methodological goals or practical use cases between these tools.

- Thank you for your comprehensive comment on comparative benchmarking and for mentioning the high standards set in this field. We completely agree on the importance of robust evaluation. We have addressed this point in detail within the manuscript, particularly in a new Appendix G. This Appendix discusses the difference between SarcGraph and specific leading tools such as SarcTrack, ZlineDetection,

SarcOptiM, and CONTRAX. Because these four tools either have different methodological goals than our updated SarcGraph or rely on data types that SarcGraph does not handle (e.g., Traction Force Microscopy data) we do not make a direct comparison with these four tools in our manuscript. However, we do make a direct comparison with two related tools: SOTA, and SarcAsM in the new Appendix G Specifically, Appendix G, along with Figure A10 and Table 2 in the manuscript, now includes a targeted comparative benchmarking against SOTA. This analysis utilizes an independent dataset published alongside the SOTA paper. It showcases the generalizability of our deep learning-based Z-disk classifier to an unseen dataset, and highlights the significant improvements of our modified SarcGraph over the 2021 version, particularly in accurate sarcomere length estimation by largely suppressing spurious detections. In addition, in Appendix G Table 3 and Fig. A11 and A12, we also compare SarcGraph with the method, SarcAsM, analyzing the quality of sarcomere detection using both approaches and providing a quantitative comparison of common features generated by both methods. Though the SarcAsM method has not been officially published in a peer reviewed journal as of re-submitting this manuscript (the SarcAsM pre-print was posted on May 4th 2025, approximately three months after we submitted this work), we believe that it is an excellent tool and therefore wanted to add a direct comparison with our method to the manuscript.

2. Computational Scalability and Performance Metrics: Providing clear, quantitative performance metrics for computational efficiency—such as runtime benchmarks on representative large-scale datasets, including details of the computing resources used—would significantly enhance the manuscript's practical relevance, particularly given its emphasis on high-throughput analysis.

- We have added a remark to the Conclusion section and included a new "Notes on Computational Performance and Scalability" section (Section 2.6) in the manuscript. This section details GPU and CPU runtime benchmarks on an NVIDIA Tesla P100–PCIe–12 GB GPU and an Intel® Xeon® E5-2680 v4 CPU, covering ensemble and single-model inference times as well as full-pipeline runtimes. Thank you for the suggestion.

Finally, figures clarity could be improved as follows:

3. Figure 2D could benefit from a clearer visual representation of the z-disc correction process, perhaps by illustrating intermediate steps explicitly.

- We appreciate the suggestion and, rather than modifying the main panel, have added Appendix E, which contains a new multi-step figure illustrating each stage of the z-disc correction pipeline in detail.

4. Figure 3C-4 should visually or textually differentiate between various scoring methods (pruning-based, z-disc probability-based, original SarcGraph scoring).

- Thank you for bringing up this point. To avoid overloading the primary figure, we've added Appendix F, where four enlarged subpanels compare the outputs of each scoring method side by side, allowing readers to see directly what structural features each emphasizes.

5. Figure 1A-iii is somewhat visually crowded; simplifying or enlarging the depicted contours would improve readability.

- We thank the reviewer for drawing attention to the clarity of the contours in this figure. In the revised manuscript we have replaced panel A-iii with a zoomed-in crop of the cell image and increased the contour line width to ensure improved readability.

6. Figure 4B captions should explicitly state the specific structural differences demonstrated between the original and modified SarcGraph results.

- We agree with the reviewer's comment and have updated the caption of Figure 4 accordingly.

7. Figures 6 and 7 could include brief annotations explaining discrepancies between predicted scores and expert scores, helping readers understand the structural features leading to these differences.

- We thank the reviewer for highlighting this. In both figures we have used red borders to draw attention to the most discrepant examples.

In conclusion, this manuscript represents an exciting methodological advancement with strong potential for wide-ranging applications. Addressing these highlighted issues will further enhance its clarity, rigor, and utility to the computational biology community.

- Thank yo so much for your encouraging feedback! We really appreciate the suggestions and have taken all of them into consideration in our revised manuscript!

Reviewer 2 Comments and Response

This is a high quality manuscript that describes new machine-learning inspired code to analyze sarcomeres and myofibrils in microscopic images. The text is very detailed and presents strengths and limitations of the proposed algorithms. The figures are impressive. The work is likely to be of substantial value to the field.

This reviewer has only on specific comment which relates to the upload to GitHub. While the source code is (likely) all there, there is not enough information in the GitHub repo to make it easy for a new user to implement the code. Including a tutorial or some worked examples with the GitHub repo would markedly increase the impact of this work.

- Thank you so much for this feedback and suggestion! We have added tutorial notebooks to the GitHub repository (GitHub repository) and made our labeled datasets and trained models available through Harvard Dataverse (Dataverse repository). The links to these resources are clearly reported in the GitHub README. We hope these additions will make the work even more impactful for new users.

Reviewer 3 Comments and Response

In this work, the authors introduce computational method to quantify the structural organization of cardiomyocytes from microscopy images. Specifically, they extend a previous computational framework to identify Z-disks and sarcomeres through a deep learning-based approach. This approach leads to better detection of Z-disks as well as integrates over multiple metrics to provide improved myofibril detection. Overall, the method leads to better characterization of structural order in cardiomyocytes, especially more disordered ones corresponding to immature/diseased samples. The study is implemented on one dataset and comparisons with expert scores are presented.

I believe the study could be of interest/use to specialists in this field, but several choices made in the methodology are unclear. Besides, it seems that the authors have missed the chance to address fundamental biological questions with their method, such as what factors does this order depend on, and how does it affect cardiomyocyte function? My specific questions are below:

Why are the features listed in section 3.5.1 the most important for sarcomere detection? Some rationalization is needed. Also, why is cell aspect ratio not considered?

- Thank you for raising these important points regarding feature selection. Regarding the features listed in Section 3.5.1: we have updated the opening sentence to clarify that these features are utilized for **quantifying cell structural organization via sarcomere properties**, rather than for sarcomere detection itself. Our primary goal in this paper is to demonstrate the feasibility of deriving a cell organization score using *only* sarcomere-centric features. Accordingly, we selected a concise set of commonly used sarcomere-based parameters from the literature (e.g., mean sarcomere length and the

orientational order parameter). Concerning the exclusion of cell aspect ratio: while it is a general morphological feature, our work specifically focuses on **sarcomere-centric quantification** of structural organization. Furthermore, the foundational study "Cell states beyond transcriptomics: Integrating structural organization and gene expression in hiPSC-derived cardiomyocytes" considered cell aspect ratio among its structural features but found it carried almost zero weight in their weighted linear model for predicting expert organization scores. This indicated its limited meaningful contribution within that established framework, aligning with our decision to omit it from our feature set given its lack of direct relevance and proven poor predictive power for the specific task.

Since sarcomeres tend to be spaced periodically (at least in the more ordered cells), an average sarcomere length can be obtained by Fourier transforming image data within a chosen sampling region. The location of the Fourier peaks directly corresponds to Z-disk spacing or sarcomere length. Can the authors compare their measurements of average sarcomere lengths with this reliable method?

- Thank you for the suggestion. In this manuscript, we focus on developing a consistent method that is applicable for quantifying the structural properties of immature and disordered iPSC-CMs – i.e., for handling the scenario where Fourier based methods fail. To this end, we extend SarcGraph, our previously developed framework, so that it more reliably detects Z-disks and sarcomeres in both mature, highly ordered cells, and in immature samples. Due to this need for generality, we do not consider Fourier transform based methods a viable alternative here – though in the revised manuscript we do make a direct comparison to the SOTA method and the SarcAsM method (see Appendix G).

In Fig. 4 (high organized case on right with improved method), this short transverse line (circled) does not seem like a true myofibril. Can the authors comment on such possible false positive detections even with the improved method?

- Thank you for drawing attention to this matter. We have updated the manuscript to address this explicitly in Figure 4 caption and section 4.1.

Some of the description of the original Sarcgraph method could be better, especially in the figures, to make this work self-contained instead of having the reader refer back to the previous method paper. For example, the authors could explain the quantities in Eq. 1 by suitably labeling fig. 3.

- Thank you for your valuable suggestion. We have now expanded Section 3.4.1 in the manuscript to provide a more detailed and self-contained explanation of the original scoring algorithm, including the definitions of the f functions and the weighting coefficients, to enhance clarity for the reader.

The entire study is based on just one dataset. Why is it convincing that the method can be reliably applied to other data sets? It would be interesting to compare for example cardiomyocyte structure in different culture conditions.

- Thank you for bringing up this valid concern. We agree that applying our method to multiple datasets would further demonstrate its broad utility. However, the hiPSC-CM image collection we used—originally published in "Cell states beyond transcriptomics: Integrating structural organization and gene expression in hiPSC-derived cardiomyocytes"—is among the few large, publicly available datasets in this field and includes both fixed (FISH) and live-imaging samples. These modalities introduce substantial variation in cell appearance and organization, providing a valid test of our pipeline's robustness. Additionally, as shown in Appendix G (Comparing SarcGraph Against Existing Methods) through comparisons with other models on different cell samples (e.g., from the SOTA associated dataset), we confirm its reliable performance. Also, we are currently evaluating the method presented in the manuscript on an independent dataset from a collaborative group, and preliminary results are very promising. Because that work is ongoing and will be reported separately within a manuscript

lead by our collaborators, we have not included it here. That being said, it did help build confidence in our method's reliable performance across diverse conditions.

There is a lot of emphasis placed in the paper on "expert scores", possibly because this has been the standard way in this field which I am not so familiar with. However, the work does not address the fundamental biological question: what does this organization depend on, and how does it matter for cell function? Factors like age, health, environment...? Can the authors correlate their cell organization scoring with the age or disease state of the cells studied, or with their function such as contractile force? Some of this information is presumably known for this published dataset, or it can be extracted from other data sets.

- Thanks for your insightful feedback. Our manuscript is built upon the framework of the original study, "Cell states beyond transcriptomics: Integrating structural organization and gene expression in hiPSC-derived cardiomyocytes," which defined expert scores as the standard measure of structural organization. The original work already extensively explored the correlations between the Cell Organization Score (COS) and factors such as cell maturity (age) and gene expression (transcript abundance) (see their Figures 5–7). Replicating these analyses is beyond the scope of our current paper. While we agree that correlating cell organization with disease status or contractile force is vital, the publicly available dataset we utilized does not provide this information. Our focus was on demonstrating the feasibility of our computational method of quantifying cell structural organization using solely sarcomere-centric features, independent of expert scores, as a foundational step for future research in exactly this direction. In future work, we will work with our experimental collaborators to address exactly these types of questions.
