# Supplementary material for: Quantifying HiPSC-CM structural organization at scale with deep learning-enhanced SarcGraph
Source: PLoS Comput Biol. 2025 Oct 3;21(10):e1013436. doi: 10.1371/journal.pcbi.1013436 (PMC12520406; doi:10.1371/journal.pcbi.1013436)
Supplement: Attachment — Submitted filename: sarcomere_review.docx [file pcbi.1013436.s002.docx]

In this work, the authors introduce computational method to quantify the structural organization of cardiomyocytes from microscopy images. Specifically, they extend a previous computational framework to identify Z-disks and sarcomeres through a deep learning-based approach. This approach leads to better detection of Z-disks as well as integrates over multiple metrics to provide improved myofibril detection. Overall, the method leads to better characterization of structural order in cardiomyocytes, especially more disordered ones corresponding to immature/diseased samples. The study is implemented on one dataset and comparisons with expert scores are presented.

I believe the study could be of interest/use to specialists in this field, but several choices made in the methodology are unclear. Besides, it seems that the authors have missed the chance to address fundamental biological questions with their method, such as what factors does this order depend on, and how does it affect cardiomyocyte function? My specific questions are below:

Why are the features listed in section 3.5.1the most important for sarcomere detection? Some rationalization is needed. Also, why is cell aspect ratio not considered?

Since sarcomeres tend to be spaced periodically (at least in the more ordered cells), an average sarcomere length can be obtained by Fourier transforming image data within a chosen sampling region. The location of the Fourier peaks directly corresponds to Z-disk spacing or sarcomere length. Can the authors compare their measurements of average sarcomere lengths with this reliable method?

In Fig. 4 (high organized case on right with improved method), this short transverse line (circled) does not seem like a true myofibril. Can the authors comment on such possible false positive detections even with the improved method?

Some of the description of the original Sarcgraph method could be better, especially in the figures, to make this work self-contained instead of having the reader refer back to the previous method paper. For example, the authors could explain the quantities in Eq. 1 by suitably labeling fig. 3.

The entire study is based on just one dataset. Why is it convincing that the method can be reliably applied to other data sets? It would be interesting to compare for example cardiomyocyte structure in different culture conditions.

There is a lot of emphasis placed in the paper on “expert scores”, possibly because this has been the standard way in this field which I am not so familiar with. However, the work does not address the fundamental biological question: what does this organization depend on, and how does it matter for cell function? Factors like age, health, environment…? Can the authors correlate their cell organization scoring with the age or disease state of the cells studied, or with their function such as contractile force? Some of this information is presumably known for this published dataset, or it can be extracted from other data sets.
